# Deafness and Sickle Cell Disease: Three Case Reports and Review of the Literature

**DOI:** 10.14740/jocmr2028w

**Published:** 2014-12-29

**Authors:** Payal Desai, Marjorie Dejoie-Brewer, Samir K. Ballas

**Affiliations:** a Division of Hematology, the James Cancer Center, the Ohio State University, Columbus, OH, USA; b Sickle Cell Disease Association, Philadelphia/Delaware Valley Chapter, Philadelphia, PA, USA; c Cardeza Foundation for Hematologic Research, Department of Internal Medicine, Jefferson Medical College, Thomas Jefferson University, Philadelphia, PA, USA

**Keywords:** Sickle cell disease, Sickle cell anemia, Hemoglobin sickle cell, Deafness, Sensory neural hearing loss, Acoustic neuroma

## Abstract

The otological complications of sickle cell disease (SCD) in general and the audiological complications in particular are not well documented and studied. Because the general management of patients with SCD has improved after the advent of newborn screening, antibiotic prophylaxis, safer blood transfusion and hydroxyurea therapy, patients with SCD are doing better in general and living longer than before. With longer longevity, the incidence of new complications of SCD became apparent and previously milder complications became more severe and more common. The dental and otological complications of SCD are examples of these changes that have become more common than before. Unfortunately with this increase, there are no guidelines or recommendations based on evidence on how to manage and treat these complications. The aim of this study was to describe three patients with SCD and deafness due to three different causes that were not adequately treated and to review the literature of deafness in SCD. We hope this may initiate more controlled trials on the incidence, prevalence and management of these complications.

## Introduction

Sickle cell disease (SCD) is a chronic complex disorder, the clinical manifestations of which are protean. Acute pain syndromes, severe anemia and its sequelae, chronic pain syndromes, infections, organ damage and various comorbidities punctuate its clinical course throughout the life span of affected patients. Among the acute pain syndromes, the vaso-occlusive crisis (VOC) is the most common and is the hallmark of the disease. Progressive organ damage may affect any organ with the brain, eyes, the pulmonary, the hepatobiliary, the spleen, the genitourinary and the musculoskeletal systems being the most commonly involved and reported. Other complications of the disease that have not been well described or studied include cranio-orbital syndromes, oropharyngeal syndromes, periodontal disease and otologic syndromes. Thus there are no established recommendations about teeth, gums, ears to discuss with patients with SCD. In this study, we report three patients with inner ear problems and review the literature that pertains to the otological complications of SCD [1-3].

## Case Reports

### Case 1

A 54-year-old African American man known to have sickle cell anemia (SS) and hypertension presented with hearing loss in the right ear of 3 months duration. The onset of hearing loss was sudden with a sensation of “water shooting up his left side of the neck”. Complications of his disease include history of leg ulcers. His last episode of VOC was greater than 10 years ago. He has no other family members with SCD or deafness. Medications include amlodipine for blood pressure control, which he has been taking since 2009, and ibuprofen as needed for pain. Physical exam demonstrates a 63 kg male in no acute distress. His blood pressure is 150/74 mm Hg. Head and neck exam is positive for mild icterus. Ear exam demonstrates a clear and mobile tympanic exam with normal clinical speech reception threshold. Orophrynx is remarkable for enlarged tonsils. His hemoglobin (Hb) is 5.0 g/dL which is at his baseline, with a platelet (PLT) count of 304 × 10^3^/μL and fetal Hb of 1.5%. Audiogram revealed normal hearing on the left side and moderate sensory-neural hearing loss with 16% discrimination on the right. MRI of the internal auditory canal revealed an enhancing lesion in the right internal auditory canal measuring 9 × 5 mm probably representing a vestibular Schwannoma (also known as an acoustic neuroma).

### Case 2

A 43-year-old deaf-mute African American man is known to have SS. Major complications of his disease, besides deaf-muteness, included frequent VOCs, priapism and leg ulcers. At the age of 18 months, when he started to speak, he developed fever of 103 °F, diarrhea, chest and abdominal pain of 2-day duration. On physical exam, he was lethargic and irritable with a stiff neck and tender swelling around the second sternal segment. Hb was 6.3 g/dL, chest X-ray showed inflammatory infiltrate over the right upper lobe and lumber puncture showed cloudy fluid with numerous gram positive diplococcic seen on smear. Blood culture and sternal aspirate also showed the presence of numerous gram positive diplococcic. Diagnosis of streptococcal septicemia with meningitis and sternal osteomyelitis was made and he was treated with intravenous ampicillin. Hematological workup showed the diagnosis of SS. He improved about 5 days after therapy with ampicillin but became deaf-mute since then. Over the years, he learned sign language with which he communicates effectively. His blood pressure has been in the normal range with the most recent being 120/84 mm Hg. His Hb in the steady state is 10 g/dL, white blood cell (WBC) count 6.8 × 10^3^ /μL, PLT 383 × 10^3^/μL and fetal Hb 5.0%.

### Case 3

A 43-year-old African American woman (Haitian ancestry) had Hb SC disease that was first diagnosed when she was 22 years old. At the time she had an episode of generalized pain that led to the diagnosis. Before that she had sporadic episodes of pain which increased in frequency and intensity during her college years. These episodes of pain were never definitively diagnosed and were thought to due to other etiologies. Besides frequent VOCs that often required treatment in the ED or hospital, she had one episode of transient ischemic attack, acute chest syndrome twice, avascular necrosis of the left hip joint and retinopathy with floaters and decreased visual acuity in the left eye. Over the years, the pain of her VOCs involved almost all areas of the body at various times with the exception of her ears. Management of her SC disease and VOCs included a strict nutritional regimen, a prescribed exercise program, complimentary medical modalities, high dose anti-inflammatories and various types of opioids with morphine being the most frequently used. Other complications included a healed superficial leg ulcer over the lower right lateral aspect of her leg at the age of 20 years and mild pulmonary hypertension.

At the age of 42 years, she had sudden hearing loss in the left ear associated with tinnitus described as the sound of a muffled speaker or a running vacuum cleaner. The tinnitus was continuous in nature without interruption. Management with steroids and ibuprofen was ineffective. Audiologic evaluations showed normal tympanic membrane, no conductive hearing loss and audiometry confirmed the diagnosis of sensorineural hearing loss (SNHL) in the left ear. Her blood pressure typically is 100/62 mm Hg, Hb 11.1 - 12.0 g/dL, WBC 6.5 × 10^3^/μL, PLT count 207 × 10^3^/μL and Hb F 2.7%.

## Discussion

Deafness is a known complication of SCD both in children and adults. It could be unilateral or bilateral, mild or severe, transient or permanent and its onset could be sudden or progressive [4-10]. More often it is due to inner ear pathology than to external ear or conductive middle ear complications [11-14]. It is rarely associated with VOCs but could be due to bacterial meningitis [4, 15, 16]. Unlike other complications of SCD, deafness and other ontological complications seem to be more common in Hb SC disease and other variants than in SS. In this paper we describe two rare clinical presentations of deafness in SS and a third patient with Hb SC disease within the framework of literature review.

The first patient described above had sudden onset of unilateral deafness that is most likely due to acoustic neuroma also known as vestibular Schwannoma. This is a benign slow-growing tumor that results from proliferation of Schwan cells covering the auditory nerves. The tumor exerts severe pressure on the balance and hearing nerve components of the eighth cranial nerve thus compromising their blood supply and causing *in situ* hypoxia. Because it exerts pressure on both the sensory and balancing nerves, it manifests itself with unilateral hearing loss and tinnitus [17-19]. Acoustic neuroma has been described once in a patient with SS. The patient was Tionne “T-Boz” Watkins, the famous singer with SS. She did have large neuroma which was treated surgically after some hesitation and fear of complications. The surgery went uneventful [17, 20]. Her case was not officially reported in a medical journal but was referred to by her surgeon, Dr. Keith Black in a book on brain surgery [17]. Thus, and to the best of our knowledge, our patient seems to be the second known patient with SS and acoustic neuroma.

The second patient we described had total deafness in both ears secondary to meningococcal meningitis. He was born in 1967; at that time newborn screening for SCD, antibiotic prophylaxis and multiple vaccinations were not available and patients with SCD were vulnerable for various types of infections. In meningitis, the infection may spread to the inner ear and irreversibly damage the hair cells of the cochlea thus causing deafness [21]. Although this patient started to speak at the age of 18 months, he lost this ability after the onset of total deafness and, hence, he became mute as well. To the best of our knowledge, this is the first patients with SS who became deaf mute due to meningococcal meningitis. However, some children who do not have SCD become deaf as a result of meningococcal meningitis [22, 23].

The third patient described above is typical of SNHL which is most common in SCD and is usually associated with vertigo, dizziness and tinnitus as common symptoms. The onset of these symptoms may be sudden with or without pain and are not associated with acute VOCs. The incidence and prevalence of these complications are age-, sex-, SCD phenotype- and geography-dependent. Systemic hypertension and impaired blood rheology due to hyperviscosity seem to be associated with otologic disorders in patients with Hb SC disease [24, 25]. The first patient, as described above, had systemic hypertension but with SS and the third patient described above had Hb SC disease with normal blood pressure but with relative hyperviscosity due to the Hb level that is > 11 g/dL which may have been a predisposing factor to deafness [25]. Three prospective, controlled, cross-sectional studies reported the effect of age on the prevalence of inner ear involvement. The first found mild SNHL in three of 80 (3.8%) Nigerian children with SS 4 - 15 years old [26]. In the second study, the SNHL occurred in seven of 52 (13.5%) Nigerian children with SS 6 - 19 years old [27]. The third study was of 167 Nigerian adults 15 - 56 years of age with SS in whom the prevalence of SNHL was 66% [28]. Similarly, in a Brazilian prospective controlled study [29], the prevalence of SNHL in 28 adults with SS was 21.4% compared to 3.6% in control subjects (P = 0.05); moreover, the prevalence was greater among patients ≥ 25 years old than younger patients (P < 0.05). In Guadeloupe (France), the prevalence of SNHL was 47.22% among patients with Hb SC disease and 43.5% among those with SS, and the majority of the patients had the Benin β^s^ haplotype [30]. The prevalence in these countries is greater than what has been reported in the UK and US [31, 32]. Moreover, in Nigeria, Brazil, and Guadeloupe (France), the prevalence of SNHL was relatively high in control groups, albeit lower than that among patients with SCD; in comparison, the prevalence of SNHL in the control group of the UK study was nil. These findings suggest that the tropics may be a predisposing factor for SNHL, possibly as a malarial effect in some countries [14, 32]. SNHL is bilateral in most patients with SCD; when it is unilateral, it is more frequent on the left side. It seems to be more common in males and in patients with Hb SC disease [26, 28, 30-32].

Due to an extensive review of available literature, it appears that the pathophysiology of inner ear complications in patients with SCD is not well understood. It appears that changes in cochlear blood flow may be the root cause of these abnormalities. However, the precise methodology pertaining to the nature of cochlear blood flow and the factors that affect it are not well known [33-35]. It is tempting to hypothesize that alterations in cochlear blood flow in patients with SCD initiate progressive local hypoxia that culminates in cellular damage and deafness. Proposed specific mechanisms of the pathophysiology of SNHL include labyrinthine hemorrhage and labyrinthitis ossificans [31, 36, 37]. What appears to be certain is that the hearing loss in patients with SS appears to be neural in nature and of earlier onset, whereas in Hb SC disease, it appears to be cochlear in nature and of later onset [30]. Labyrinthine hemorrhage is usually associated with sudden onset of SNHL and vestibular syndromes, such as tinnitus, vertigo, and dizziness, and seems to be more common in Hb SC disease than in SS [31, 37]. Labyrinthitis ossificans is associated mostly with SNHL and may be the result of inflammation secondary to vaso-occlusion [36].These complications appear to be associated with specific anatomic structures of the inner ear, with labyrinthitis ossificans always identified in the lateral semicircular canal and hemorrhage in the basal turn of the cochlea and vestibule [31]. Patients with SCD who complain of inner ear symptoms should have a thorough workup including computed tomography and MRI.

In summary, the otologic complications of SCD in general and deafness in particular are not well understood and poorly studied. Controlled studies about the incidence, prevalence, clinical manifestations and pathophysiology of these complications in SCD are needed.
